# Surgical skills simulation in trauma and orthopaedic training

**DOI:** 10.1186/s13018-014-0126-z

**Published:** 2014-12-19

**Authors:** Euan RB Stirling, Thomas L Lewis, Nicholas A Ferran

**Affiliations:** Orthopaedic Department, Northampton General Hospital, Cliftonville, Northampton, NN1 5BD UK; General Surgery, Kingston Hospital, Galsworthy Road, Kingston-upon-Thames, KT2 7QB UK; Shoulder Fellow, Prince of Wales Private Hospital, Randwick, NSW 2031 Australia

**Keywords:** Simulation, Training, Orthopaedics, Trauma, Medical education, Surgical education, Clinical competence, Computer simulation, Arthroscopy

## Abstract

Changing patterns of health care delivery and the rapid evolution of orthopaedic surgical techniques have made it increasingly difficult for trainees to develop expertise in their craft. Working hour restrictions and a drive towards senior led care demands that proficiency be gained in a shorter period of time whilst requiring a greater skill set than that in the past. The resulting conflict between service provision and training has necessitated the development of alternative methods in order to compensate for the reduction in ‘hands-on’ experience. Simulation training provides the opportunity to develop surgical skills in a controlled environment whilst minimising risks to patient safety, operating theatre usage and financial expenditure. Many options for simulation exist within orthopaedics from cadaveric or prosthetic models, to arthroscopic simulators, to advanced virtual reality and three-dimensional software tools. There are limitations to this form of training, but it has significant potential for trainees to achieve competence in procedures prior to real-life practice. The evidence for its direct transferability to operating theatre performance is limited but there are clear benefits such as increasing trainee confidence and familiarity with equipment. With progressively improving methods of simulation available, it is likely to become more important in the ongoing and future training and assessment of orthopaedic surgeons.

## Introduction

Surgical training has evolved considerably from the historical apprenticeship of the early surgeons to a complex structured training programme with multiple assessments. As trainee numbers increase, opportunities to develop procedural and technical skills become increasingly limited. Furthermore, introduction of the European Working Time Directive (EWTD) across Europe [1] and the 80 h per week limit introduced by the Accreditation Council for Graduate Medical Education (ACGME) in the United States of America [2] have led to a reduction in training hours available during the designated training period. As a result, there has been a continued reduction in opportunities for ‘hands-on’ experience for trainees across all surgical specialties. Senior delivered care and a move to limit ‘out of hours’ operating have further reduced the opportunity for independent surgical experience for trainees, whilst procedures traditionally performed by junior doctors are now often the remit of their senior colleagues. Orthopaedic surgery is no exception. These constraints have forced an adjustment to a more focused, competency-based assessment of proficiency, compared with a previous assumption of this through experience.

In the same period, there has been a significant development of novel techniques requiring current trainees to master a greater array of specialist skills, despite having less time in which to do so. Innovation has meant that there are more occasions where surgical intervention may be indicated. This, coupled with an ageing population more expectant of treatment, has resulted in greater demand for surgery than ever before leading to a conflict between service provision and training.

Reform is required to ensure that current surgical trainees are competent and efficient enough to act autonomously on completion of training and have developed the necessary expertise to subsequently deliver instruction themselves. Many different solutions including e-Learning, simulation and compulsory fellowship training programmes have been proposed to maximise learning opportunities within existing resource constraints. One such solution, simulation, allows trainees to practice skills in a safe controlled environment and has been shown to improve confidence whilst minimising patient risk [3]. The definition of simulation in medicine can be broadly defined as “any technology or process that recreates a contextual background in a way that allows a learner to experience mistakes and receive feedback in a safe environment” [4]*.* This definition is constantly evolving due to improvements in technology which allow increasingly complex situations to be modelled and tested. Simulation aims to recreate the experience of patient care without compromising patient safety. The ability to modify a situation allows trainees to experience novel but often important situations that may not be commonly experienced in clinical practice. The benefits of simulation are well recognised in many other specialties including general surgery [5], emergency medicine [6] and anaesthetics [7], and it has been advocated by many of the governing medical bodies and Royal Colleges in the United Kingdom [8–10]. The advantages of simulation extend beyond simple technical and procedural skills. Simulation allows trainees to engage with a multi-disciplinary team and focus on individual and team-based cognitive skills including problem solving, decision-making, and team behaviour skills.

Within surgery, simulation is not a new concept, as cadaver models were historically used as part of surgical training. However, in recent decades, significant progress has been made in developing new and varied simulation-based techniques to provide training in a safe and modifiable environment [11–13]. We review the different methods available within orthopaedic surgery and the available evidence supporting their use. A summary of simulation models currently available within orthopaedic surgery and their respective advantages and disadvantages can be seen in Table 1.

**Table 1:** **A summary of the main simulation modalities available to orthopaedic surgery trainees**

**Simulation model**	**Advantages**	**Disadvantages**
Cadaveric simulation		Expensive
	High fidelity	Not easily accessible with specialist storage demands
		Time-consuming preparation time
	Shown to develop transferable operative skills	Relies on tissue donation
		Risk of disease transmission
	Allows understanding of relevant clinical anatomy and surgical approaches	Lack of uniformity amongst specimens
Synthetic bone simulation	Relatively inexpensive, portable and widely available	
	Widely available	Does not allow understanding of influence of soft tissues
	Develop understanding and familiarity with orthopaedic instruments and equipment	Lack of true haptic feedback
Arthroscopic simulation	Able to record progress and assess motion analysis	
	Allows for development of hand-eye co-ordination and triangulation	High initial setup costs
	Wide range of procedures may be possible	Limited realism
	Modern simulators can provide haptic feedback	
Virtual reality simulation	Able to record progress and assess motion analysis	
	Wide range of procedures may be possible	High initial setup costs
	Allows for scenario simulation	
Cognitive simulation	Potentially cost free	Limited evidence to support use in clinical training/improvement in technical procedural skills
	Accessible on mobile devices	
	Point of care education	

### Simulation in orthopaedic surgery

Orthopaedic surgery lends itself to simulation training better than many other medical or surgical specialties due to consistency in skeletal anatomy and has been employed in various forms for decades. The Arbeitsgemeinschaft für Osteosynthesefragen (AO) foundation has been delivering training in basic fracture management using synthetic bones for more than 50 years in over 100 countries and has since developed many more complex instructional courses [14]. The advent of minimally invasive arthroscopic surgery has demanded a further subset of skills, which again can be well practised in a simulated environment. Advances in computer-simulated technology allows for increasingly realistic recreation of clinical scenarios without risks to patient safety. Simulation is likely to become an increasingly utilised method of gaining experience, with new trainees being exposed to it from as early as medical school. The current curriculum for specialist training in orthopaedics in the United Kingdom reflects this, identifying three different pathways for simulation in recognition of its possible benefits [15].

The growing role of simulation within surgical training has demanded that tools be developed to allow objective evaluation of the technical skills learnt in order to validate its use. A number of assessment tools for surgical simulation have been developed including the Objective Structured Assessment of Technical Skills (OSATS) and Global Rating Scale of Performance (GRS). These systems both rely on the same principle of scoring against preset criteria by trained assessors. Specifically, the OSATS checklist consists of a set of manoeuvres deemed to be essential elements of a procedure, whilst the GRS focuses on specific surgical behaviours [16]. These have been shown to reduce the biases associated with direct observation by experts alone [17,18]. Novel simulation tools including the use of computers, virtual reality and cognitive task simulation offer the potential for advanced data assessment based on user input. The use of alternative assessment techniques including motion tracking and video assessment has been shown to reliably and objectively correlate to surgical performance although there is limited data to support this on a wider scale [19,20]. Howells et al. divided 35 subjects into a surgeons group (*n* = 20) and a non-surgeons group (*n* = 15). The surgeons group was further subdivided based on the amount of previous arthroscopic experience. Each participant performed two separate simulated arthroscopic tasks whilst being assessed with motion analysis equipment. The time taken, total path length and number of movements were recorded with a significant difference in performance identified between surgeons and non-surgeons (*P* < .0001) and between senior and junior surgeons (*P* < .05). Tashiro et al. tested 12 surgical trainees, 12 orthopaedic residents and 6 experienced arthroscopic surgeons on a synthetic bone knee simulator. Subjects performed a joint inspection and probing task and a partial meniscectomy task whilst measuring the trajectory data and force data. The experienced group performed both tasks with higher scores and more quickly than the less experienced groups.

### Cadaveric simulation

**Figure 1:**
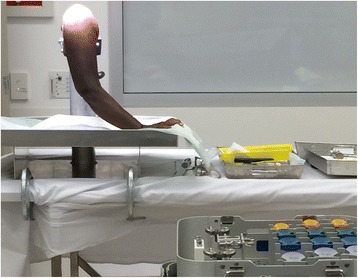
**Cadaveric upper limb workshop.**

Cadaveric practice has been employed in surgical training for centuries and remains a highly regarded method of training due to the exposure to real anatomy and indeed anatomical variation (Figure 1). Furthermore, it allows the trainee to appreciate planes of dissection and practice soft tissue handling. In recognition of this, The Royal College of Surgeons of England has established The Wolfson Centre, one of the world’s most advanced cadaveric dissection facilities, where numerous orthopaedic courses are held.

Evidence suggests that cadaveric training is of benefit in reducing error prior to real-life operation. A study on the placement of cadaveric thoracic spine pedicle screws demonstrated a reduced technical error rate with increased practice of the procedure and experience of the surgeon [21]. Surgical error rate of pedicle screw placement was assessed as novice surgeons placed pedicle screws on five consecutive cadaveric spines. Initially, surgical error rate was high; however, there was a significant decrease in the proportion of total surgical errors by the third cadaver and a significant decrease in critical surgical errors by the fourth cadaver. However, although strict objective measures were taken, the study size was small (three candidates) and there was no control group. Anastakis et al. [22] demonstrated improved performance of six surgical procedures, including flexor tendon repair and K-wire fixation of a metacarpal fracture, assessed on cadaver models when cadaver or bench model training had been given compared with text learning. However, there was no significant difference in competence between groups trained on cadaver and bench models. Nonetheless, they concluded a strong possible transfer to performance in theatre.

There are however multiple drawbacks to cadaveric training. Preparation and storage of specimens confers a significant time and financial cost. It relies upon donations, which are limited, and it is therefore vital that specimens are used for training in a way that will provide the greatest benefit. Perhaps of greatest significance is the lack of direct evidence relating simulated cadaveric techniques with *in vivo* operating performance.

### Synthetic bone simulation

**Figure 2:**
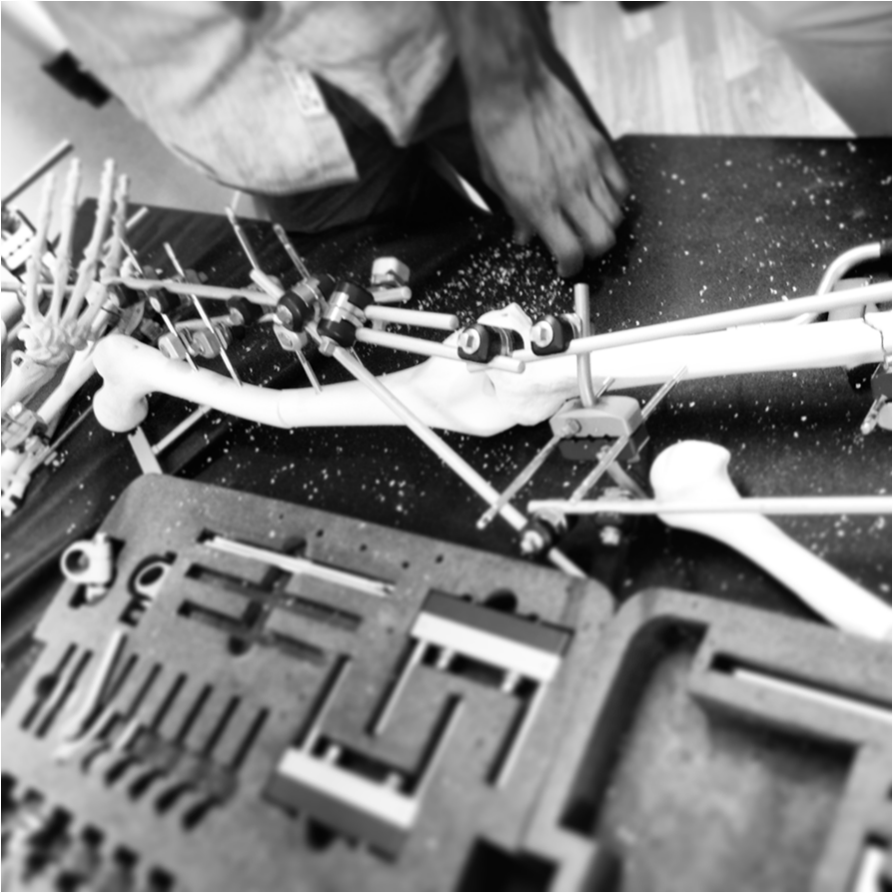
**Synthetic bone fracture fixation workshop.**

Practice of orthopaedic surgical techniques using synthetic bone substitutes has acted as a mainstay of training throughout the second half of the 20th century and beyond (Figure 2). Consistency of skeletal anatomy allows for easy production of replica bones in large numbers for minimal cost, on which basic fracture fixation skills may be practiced without risk to patients. Furthermore, there are minimal associated storage costs, and ethical approval is not required for their use. However, despite modifiable characteristics, these replicas lack the unique internal architecture and viscoelastic properties of a real bone [23]. Training using this method may therefore allow development of ability in a particular technique but lack the ‘true feel’ of a real bone. Frequently, this type of simulation is limited to the practice of bone handling and fixation in isolation, without consideration of soft tissue anatomy, and therefore lacks realism.

As discussed, there is evidence that there is no difference between cadaveric and bench model training prior to assessment of fixation of metacarpal fracture on a cadaver [22]. However, this compared only Kirschner-wire (K-wire) fixation and therefore might not have allowed discrimination between real and synthetic bone in the same way that a more complex technique would. As an example, Leong et al. [24] compared three different models of fracture fixation as an assessment of technical skill in varying grades of surgeon. The first assessment involved application of a dynamic compression plate to a cadaver porcine model, whilst the others assessed insertion of an unreamed tibial intramedullary nail and application of a forearm external fixator to synthetic bone. Measured using the OSATS checklist and a GRS, the results demonstrated a significant difference in the performance on the application of the dynamic compression plate between the grades of surgeon, but no significant disparity in results on the synthetic bone models. This suggests that although synthetic bone models may benefit junior trainees in developing experience of the techniques and instruments used, they may lack the fidelity to be of value to more senior surgeons. A recent small study by Yanping et al. found that the use of haptic feedback combined with a synthetic bone simulator significantly improved a trainee’s bone sawing skill in the field of maxillofacial surgery [25].

### Arthroscopic simulation

Arthroscopy is one of the most commonly performed orthopaedic procedures, with an ever-increasing number of indications and therapeutic options available. Benefits of arthroscopic procedures are well known and include shorter recovery time, reduced risk of infection and option to perform in an outpatient setting. It has become a key component of practice and a core skill of orthopaedic training [11,12,15,26]. However, development of arthroscopic skills takes considerable time and is associated with a significant financial burden when this training takes place in the operating theatre [27]. Furthermore, there is an increased risk of iatrogenic injury during early arthroscopic training using the traditional apprenticeship model [28,29].

Arthroscopy is therefore well suited to simulation training. It presents unique technical challenges, namely, the concurrent interpretation of proprioceptive and visual stimuli from a three-dimensional structure presented as a two-dimensional image and development of competence in triangulation. These skills are best acquired through actual instrument handling and rely on realistic substitutes for live patients [30]. Cadaveric models have traditionally been used [31], but the drawbacks already discussed remain. More recently, simulation has been practised on bench models and virtual reality (VR) systems, which allows rehearsal of a surgical procedure in a virtual three-dimensional environment. Similar concerns of the fidelity of these models and their transferability to the operating theatre exist as with simulation of open procedures. There have been promising advances in technology, in particular development of haptic simulation, where tactile feedback is given to the operator by generation of intermittent artificial mechanical resistance, which have improved realism. However, there are no studies to date demonstrating a benefit of haptic simulators compared to non-haptic ones [16]. There is nonetheless an undoubted advantage of virtual reality systems, an inbuilt mechanism of recording progress, through measurements such as motion analysis and number of probe collisions [32].

Laboratory-based bench models are available which allow practice of arthroscopic procedures on bone and plastic models using real arthroscopic stacks and equipment. This develops familiarity with equipment and allows various procedures to be attempted such as meniscectomy, tissue debridement and rotator cuff repair without risk of patient morbidity or loss of theatre operating time. Experience of this type has been shown to transfer well to the operating theatre. Howells et al. [33] randomised 20 orthopaedic trainees to receive a fixed protocol of arthroscopic simulator training on a benchtop knee model or no additional training. Diagnostic knee arthroscopy was then assessed in theatre following traditional instruction and demonstration. Performance of the intervention group was significantly better than the untrained group as assessed by the intra-operative technique section of the procedure-based assessment for diagnostic arthroscopy from the Orthopaedic Competence Assessment Project (OCAP) [34] score and a 5-point GRS. There is evidence that repeated simulated practice of an arthroscopic skill is beneficial. A further study by Howells et al. [35] showed a statistically significant improvement in the ability to perform arthroscopic Bankart repair sutures on an Alex Shoulder Professor benchtop simulator with repeated experience. Six consultant orthopaedic surgeons specialising in lower limb surgery viewed an instructional video demonstrating the technique. Each then performed the procedure on four occasions over a period of weeks. Grading was performed by the supervising authors, using a fixed diameter arthroscopic hook, to check for gapping between the labrum and the glenoid and for knot strength. Further assessment was provided using a validated [19] three-dimensional electromagnetic motion tracking system. There was a statistically significant improvement in path length, number of hand movements and time taken between initial and final attempts demonstrating a clear learning curve. The study was repeated 6 months later, after no further training, with similar results. However, there was no significant difference between results in the initial and repeat study suggesting that there was minimal retention of the previously acquired improved skill level. This strongly advocates the use of simulation as a means of developing technical ability before *in vivo* practice but also provides evidence that a skill may be lost if not routinely used. Jackson et al. [36] recorded similar outcomes of a clear improvement in performing simulated arthroscopic meniscal repair on a knee simulator over a 3-week period as assessed by time taken, distance travelled and number of hand movements. In contrast to Howells et al., there was no significant loss of skill after a 6-month interval. Despite the clear fidelity of this training method, there are drawbacks. Assessment relies on supervision and feedback from senior faculty as there is often no inbuilt mechanism of recording progress as found in VR models [32].

Multiple studies have attempted to validate VR arthroscopic simulators by demonstrating a correlation between real-life arthroscopic experience and performance on a simulator [11,26,36–38]. Experienced surgeons have achieved better results in performing VR-simulated arthroscopic tasks as measured by one or a combination of time to complete a procedure, computer-assessed motion analysis compared with a predetermined optimum and number of probe collisions [29,30,39–41]. Gomoll et al. performed a follow-up study of arthroscopic ability of 10 orthopaedic trainees, assessed on the same simulator 3 years after initial testing, and showed a significant improvement in performance [42]. The inference from this, that simulation can be beneficial to training given its ability to distinguish between surgeons of different grades, is endorsed by questionnaire assessment of participants. Tuijthof et al. [30] measured face validity, educational value and user-friendliness of two simulators through a 10-point numerical rating scale following completion of a simulated task. Validity was found to be sufficient, but not perfect for both simulators, confirming the need for continued improvement of the models. However, all participants, novice, intermediate and expert surgeons, felt that there was definite educational benefit from the systems used.

Evidence demonstrating improved arthroscopic ability following simulated training also exists. Andersen et al. [43] randomised 14 inexperienced surgeons into intervention and control groups for testing on a VR arthroscopic trainer with the intervention group receiving 5 h of pre-assessment training. A second control group of experienced surgeons was also tested. The intervention group demonstrated a significant reduction in time to task completion, distance travelled by the camera and depth of collision compared with the inexperienced control group. However, this does not provide any evidence for performance in a real surgical setting. Martin et al. [44] reported a correlation between task performance on a simulator model with subsequent performance in a cadaver model. Task completion time on the simulator was found to be a significant predictor of time completion on the cadaver model. There is, to date, no evidence demonstrating transferability of VR arthroscopic skill to the operating theatre, and this presents the next step in evidence for this training method.

### Virtual reality simulation

The rapid advancements in computer technology and imaging over recent decades have opened up a new method for surgical simulation. Indeed, many systems are sufficiently developed that they are frequently used by practising surgeons for pre-operative planning. Rehearsal of procedures is now possible using mobile simulation software applications [45]. As with synthetic models, the relative reproducibility of skeletal anatomy makes this an effective tool. An additional benefit of these tools is the feasibility of recreating various surgical scenarios, for example different fracture patterns, without the need for any new equipment. Thus, after an initial outlay, it becomes a relatively cheap training option. Furthermore, repeated attempts at a procedure can easily be made in a safe environment with immediate feedback often possible when built in to the simulator.

Software tools have been developed which allow a complete surgical procedure to be practised in a virtual environment. Blyth et al. [46] reviewed a personal computer (PC)-based virtual reality training system allowing simulation and assessment of hip fracture fixation. The simulator presents different scenarios of increasing difficulty of fracture fixation on a virtual hip model with two-dimensional radiographic images used to guide fracture reduction and implant placement. Ten participants with differing levels of experience performed six scenarios before completing a 26-part questionnaire to assess the face validity of the simulator. The results demonstrated that the simulator was both realistic and also tested problem-solving ability. The fidelity of the simulator was reinforced in its ability to differentiate between surgeons with different levels of experience [47]. There was a statistically significant difference in the accuracy of procedure, number of X-rays needed and speed between novices (medical students) and trainee surgeons.

**Figure 3:**
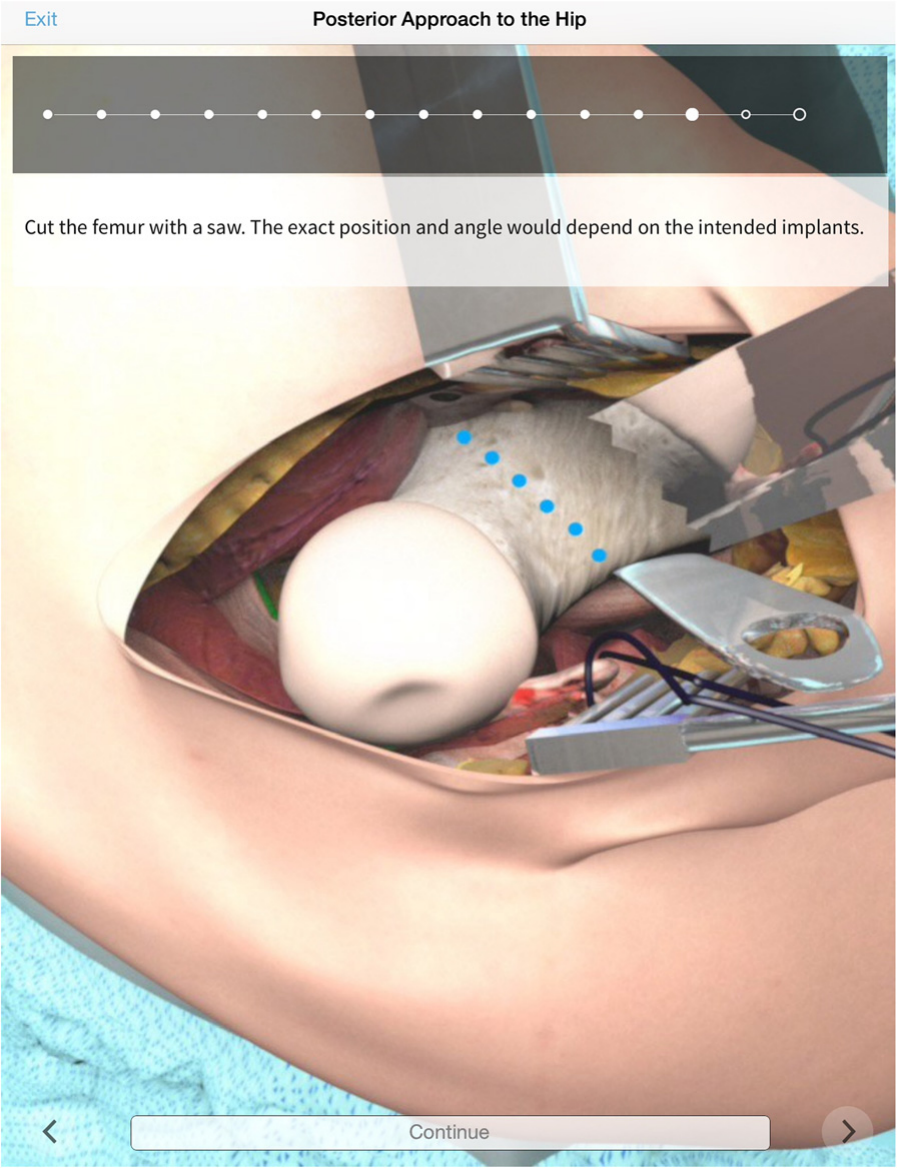
**Screenshot of “Touch Surgery” app module on posterior approach to the hip.**

Advances in mobile computing technology have allowed the development of mobile software applications or ‘apps’, which allow trainees to simulate the various stages of an operation or review relevant intra-operative information. These apps utilise photo-realistic graphics and decision-making software to provide an engaging virtual operative experience. One such app is ‘Touch Surgery’, which allows users to simulate over 30 common operations on a mobile device, independent of time or geographic location [48] (Figure 3). Whilst these mobile apps do not allow development of physical surgical skills, they do allow trainees to cognitively simulate the stages of each operation, thus building an awareness of potential complications. Given the recent development of these apps in the context of mobile simulation, there is no evidence to support or oppose their use for surgical skills simulation.

**Figure 4:**
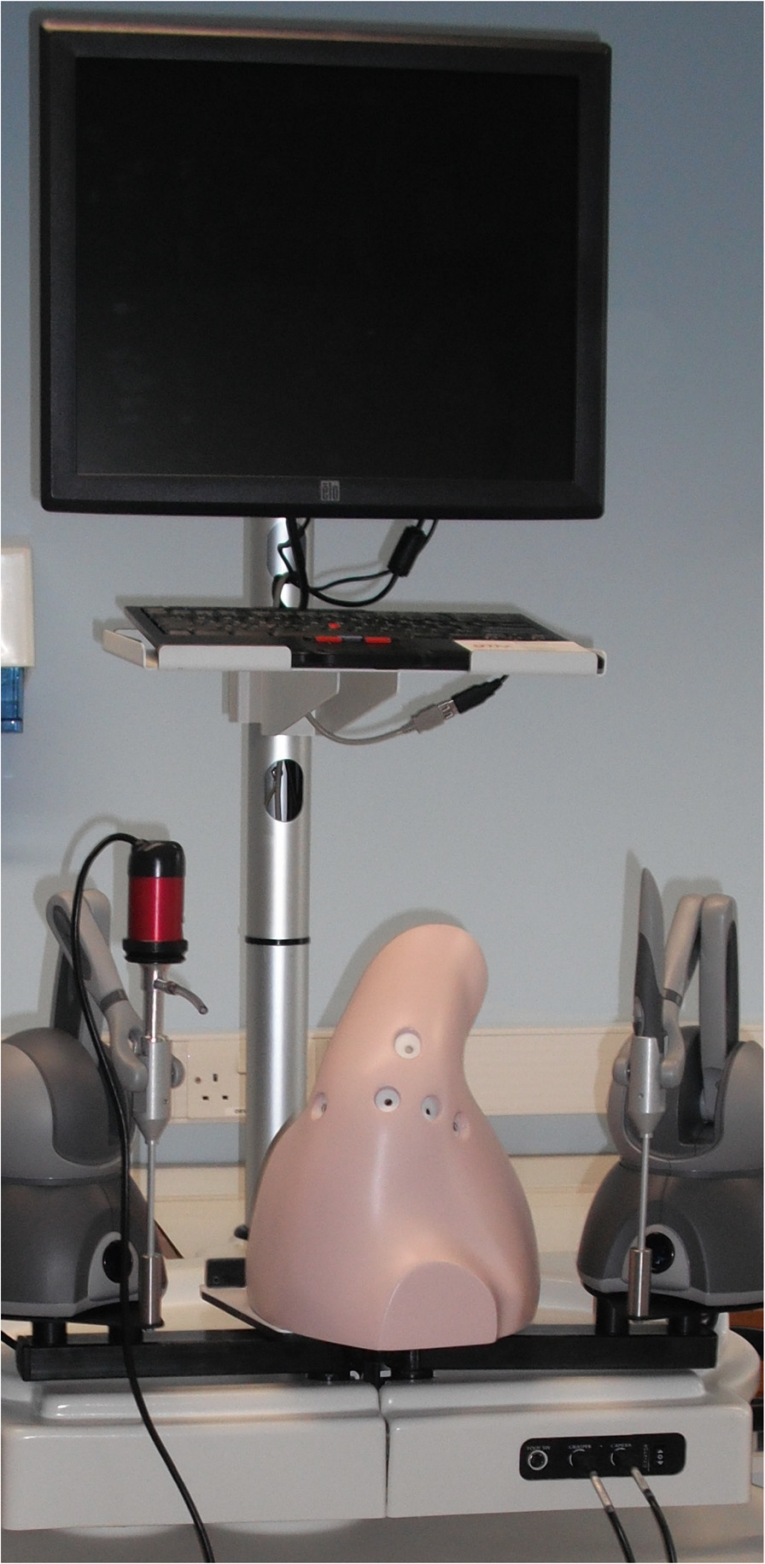
**Virtual Reality arthroscopic simulator with haptic feedback.** (Insight Arthro VR, Simbionics USA).

Despite the improvements in technology and particularly the introduction of haptics to simulation, uncertainty remains about its fidelity compared with more traditional methods (Figure 4). LeBlanc et al. [49] compared simulated surgical fixation of the ulna by 22 orthopaedic residents using a synthetic bone simulator and a VR haptics system. Participants were assessed on both models by experienced examiners familiar with the task and by time to completion. The results demonstrated construct validity of the systems, with both simulators differentiating between different grades of surgeon. However, there was no significant correlation in the performance of participants between the two simulators; therefore, concurrent validity was not achieved. This suggests that although the VR system may help trainees to learn and develop basic skills, it may not be as effective as a synthetic bone model. Continued development in these systems is required, and improvements may yet build on the initial promise shown.

### Cognitive simulation

Cognitive simulation is one of the newest examples of innovation within surgical training. It is the process whereby trainees assess and rehearse actions within their mind without physical movement. It is hypothesised that trainees may improve their intra-operative performance and surgical skills through appropriate pre-operative cognitive simulation, either with or without appropriate aide memoires. These techniques have been used to great success in other domains including elite sport [50]. Evidence of transference to practical surgical ability to date remains limited; however, it is an exciting prospective new technique, which may become integral to future training.

Shiralkar states that there is little difference between real and imagined experiences provided that the experience is imagined in a specific manner [51]. Evidence suggests that similar neural pathways are stimulated from imagined muscle movements as physical ones and therefore may be as effective as physical practice [52]. The application of this technique in surgical training is obvious. If proven to be effective, it would provide a low-cost, easily accessible tool that can be applied to multiple different procedures without the need for specialist equipment. The Association of Surgeons in Training (ASiT) in the United Kingdom has recognised the potential and cognitive simulation courses have been delivered with positive feedback [53].

Currently, the literature offers little direct evidence of whether cognitive simulation can improve theatre performance of current orthopaedic surgeons in training or indeed consultants learning a new technique. Kohls-Gatzoulis et al. [54] performed a prospective study of the ability to correctly execute total knee arthroplasty assessed using OSATS method, an error detection exam and a post-course multiple choice question (MCQ) exam. Junior surgical residents were randomised into two groups, one focusing on technical skills with more opportunity to practice the task. The other aimed to develop cognitive skills, and physical practice was more limited. There was equivalence in the OSATS scores and post-course MCQ between the groups, but the cognitive skills group achieved statistically significant better scores on the error detection test suggesting that this is a potentially useful technique to introduce into training.

Although assessment of transferability of this technique will be difficult to accurately assess, it is the most easily accessible training method discussed. Furthermore, it is a promising technique as it does not simply take a procedure in isolation and is perhaps the only method of simulation available that can recreate all the different components involved in surgical practice together such as pre-operative planning, technical skill and communication. Further investigation of its role is therefore warranted.

## Conclusion

There is no doubt that simulation training has a significant role to play in current and future orthopaedic training, and this is likely to increase further. There will be continued advances in technology to improve realism and increased availability of simulators, which may help to compensate for the reduced real-time theatre experience of current surgeons in training.

The aviation industry has led the way in demonstrating the advantages of simulation, and it forms a key component of training and continuing assessment throughout a pilot’s career. There is little doubt that similar benefits can be gained in surgical practice with direct improvements to patient safety. The medical literature certainly suggests a significant benefit of simulation for improving trainee confidence and understanding of techniques whilst also allowing practice and development of specific technical skills. However, there remains limited evidence for the value of simulation in its transferability to proficiency in the operating theatre. A further possible drawback of this type of training is its focus on the technical aspect of surgery in isolation. Real surgical practice is an inherently demanding task, and even the most validated training simulators will not be able to recreate all the different components that an operating surgeon must manage. The fidelity of surgical simulators will remain one of the biggest challenges as their use becomes more widespread, with continued development needed as new procedures are developed. This requires focus on the technical components of a model of simulation but also a greater emphasis on the integration of the other elements of a surgical procedure such as pre-operative planning and consent, intra-operative communication and consideration of alternative management options if required. This will ensure that it becomes a truly useful adjunct to training.

However, it is vital that both trainees and trainers do not forget that technical ability forms only one component of the skill set required to be an accomplished surgeon. Inherently, leadership and communication skills are required in surgical practice and in certain circumstances may be of greater importance than technical aptitude. Training methods must therefore address this and develop these skills alongside procedural learning. Further research into surgical simulation should focus on the impact of simulation training on patient safety, the transfer of skills into practical theatre settings and further validation of the simulation tools for procedural competency. A recent editorial in The Bone and Joint Journal News states that ‘a good surgeon needs head, hand and heart’ [55]. Whilst simulation may facilitate in the challenge of gaining sufficient technical aptitude, it ‘fails to address the two other essential facets, clinical experience and attitude’.

## Abbreviations

EWTD: European Working Time Directive
ACGME: Accreditation Council for Graduate Medical Education
AO: Arbeitsgemeinschaft für Osteosynthesefragen
OSATS: Objective structured assessment of technical skills
GRS: Global rating skill
K-wire: Kirschner wire
VR: Virtual reality
OCAP: Orthopaedic Competence Assessment Project
PC: Personal computer
ASiT: Association of Surgeons in Training
MCQ: Multiple choice question
